# Optimizing the Efficacy–Toxicity Paradigm in Pediatric Oncology: A Narrative Review of Immunotherapy and Survivorship Outcomes

**DOI:** 10.3390/curroncol33050298

**Published:** 2026-05-20

**Authors:** Zaure Dushimova, Timur Saliev, Aigul Bazarbayeva, Kymbat Karimova, Abay Kussainov, Ildar Fakhradiyev

**Affiliations:** 1 Department of Fundamental Medicine, Al-Farabi Kazakh National University, Almaty 050040, Kazakhstan; zaure.dushimova@kaznu.edu.kz; 2 Institute for Fundamental and Applied Medical Research, S.D. Asfendiyarov Kazakh National Medical University, Tole Bi Street 94, Almaty 050000, Kazakhstan; 3 Scientific Centre of Pediatric and Pediatric Surgery, Al-Farabi Avenue 146, Almaty 050060, Kazakhstan; 4 College of Medicine, Korea University, Seoul 02841, Republic of Korea

**Keywords:** pediatric, leukemia, immunotherapy, relapse prevention, blinatumomab, neuroblastoma

## Abstract

Background: Childhood cancer survival now approaches 80% in high-income countries, yet most survivors face lifelong toxicity. This review examines the interplay between treatment efficacy, relapse prevention, and therapy-related complications. Methods: Narrative synthesis of landmark pediatric oncology trials (2000–2026), including AALL1731 (blinatumomab), ELIANA/PLAT-02 (CAR T-cell), and GD2-CART01 (neuroblastoma), with comparative analysis of efficacy and toxicity. Results: In AALL1731, adding blinatumomab to chemotherapy improved 3-year disease-free survival from 87.9% to 96.0% (HR = 0.39, 95% CI: 0.27–0.56, *p* < 0.001), but increased sepsis from 5.1% to 14.8%. Comparison between AALL1731 (front-line blinatumomab) and ELIANA (CAR T-cell in relapsed disease) reveals that earlier immunotherapy deployment yields better outcomes: 96% DFS vs. 48% 3-year EFS, respectively. In GD2-CART01, early use (after 1–2 prior lines) achieved 89% 5-year survival vs. 43% with delayed use (HR = 0.31). Approximately 95% of survivors experience ≥1 late effect, with 60–90% carrying chronic conditions into adulthood. Conclusions: Immunotherapy transforms outcomes, but timing is critical, as earlier deployment dramatically improves survival. Toxicity remains pervasive, requiring systematic mitigation strategies.

## Simple Summary

At present, nearly 80% of children diagnosed with cancer are now cured, representing remarkable therapeutic progress. Yet most survivors face lifelong health challenges, including heart damage, infertility, secondary cancers, and cognitive impairment. This review examines how novel immunotherapies, such as blinatumomab for leukemia and CAR T-cells for neuroblastoma, dramatically improve survival while introducing distinct toxicities. A key finding is that treatment timing critically influences outcomes: earlier use of these therapies yields significantly better results. These insights underscore that true success in pediatric oncology extends beyond achieving a cure to minimizing treatment-related harm, ensuring that children not only survive but thrive long after therapy ends.

## 1. Introduction

The landscape of pediatric oncology has transformed beyond recognition over the past five decades. What was once a near-certain fatal outcome for most children diagnosed with cancer has transformed into a disease with 80% overall survival in high-income countries [1,2]. This remarkable progress represents one of modern medicine’s greatest achievements, a testament to the power of collaborative clinical trials, incremental therapeutic optimization, and unwavering commitment to improving outcomes for the most vulnerable patients.

However, this success has revealed a fundamental limitation: survival does not equate to health. Approximately 95% of childhood cancer survivors experience at least one treatment-related health issue. The intensive multimodal therapies that cure cancer include multi-agent chemotherapy, radiation, surgery, and increasingly immunotherapy [3,4]. Approximately 95% of childhood cancer survivors will experience at least one health-related issue related to their prior treatment during their lifetime, with 60–90% carrying at least one chronic health condition into adulthood [5,6]. The child cured of cancer at age five may face cardiomyopathy at thirty, secondary malignancy at forty, and cognitive decline that affects educational attainment, employment, and independent living throughout the lifespan [7].

This dual dichotomy, extraordinary progress alongside persistent and often devastating toxicity, defines the central challenge of contemporary pediatric oncology. The tension facing pediatric oncologists today is no longer simply about extending life, but about doing so while preserving its quality [8]. This requires a fundamental rethinking of what constitutes treatment success, moving beyond the traditional metric of five-year survival to encompass the full spectrum of outcomes that matter to patients and families: freedom from relapse, minimization of acute toxicity, preservation of organ function, maintenance of neurocognitive health, and the ability to live a full and productive life after cancer.

Conventional cytotoxic chemotherapy has reached a biological ceiling: further dose intensification produces diminishing returns in efficacy while causing unacceptable acute and long-term toxicity. This limit, defined by myelosuppression, organ toxicity, and secondary malignancies, has necessitated a paradigm shift toward targeted immunotherapies that harness the patient’s own immune system to eliminate malignant cells with greater specificity.

The pathophysiological basis of late treatment toxicity involves multiple molecular mechanisms. Anthracyclines, for example, generate reactive oxygen species that cause mitochondrial DNA damage and cardiomyocyte apoptosis, leading to progressive cardiac dysfunction that may manifest decades after treatment. Alkylating agents induce DNA crosslinks that, when repaired incorrectly, promote genomic instability and secondary malignancies. Cranial radiation triggers chronic neuroinflammation and oxidative damage to neural progenitor cells, resulting in cognitive decline. Understanding these mechanisms is essential for developing mitigation strategies.

This review examines how the interplay between treatment efficacy, relapse prevention, and therapy-related toxicity analysis has shaped contemporary approaches to improving survival in childhood malignancies. It draws upon lessons from landmark clinical trials that have transformed practice, the addition of blinatumomab to standard-risk ALL therapy, the development of GD2-directed CAR T-cells for neuroblastoma, and the molecularly informed risk stratification of medulloblastoma, while simultaneously confronting the sobering reality of relapse and the hidden burden of treatment-related toxicity [9]. It explores how analysis of treatment failure reveals weaknesses in current approaches and illuminates pathways for improvement. It examines the toxicity burden as the hidden determinant of survival, whose understanding is essential to achieving not just a cure but cure with quality. In addition, it identifies critical knowledge gaps that must be addressed to ensure that the next generation of children with cancer achieves not only higher cure rates but also healthier, fuller lives beyond their disease.

The central thesis is clear: sustainable improvements in pediatric cancer survival depend not only on developing more effective therapies but equally on understanding and mitigating the complications that accompany treatment. By analyzing treatment outcomes and therapy-related toxicities, clinicians and researchers have learned that the two are inextricably linked, excessive toxicity limits treatment intensity.

## 2. Methods

### Search Strategy

A systematic literature search was conducted using PubMed/MEDLINE, ClinicalTrials.gov, and the Cochrane Library (1 January 2000–28 February 2026). The following search strings with Boolean operators were used:

For leukemia immunotherapy: (“pediatric ALL” OR “acute lymphoblastic leukemia” OR “B-ALL”) AND (“blinatumomab” OR “CAR T-cell” OR “tisagenlecleucel”) AND (“efficacy” OR “survival” OR “relapse” OR “toxicity”).

For solid tumors: (“neuroblastoma” OR “pediatric solid tumor”) AND (“GD2” OR “dinutuximab” OR “CAR T-cell”) AND (“immunotherapy” OR “targeted therapy”).

For toxicity/late effects: (“childhood cancer survivor” OR “pediatric oncology late effects”) AND (“cardiotoxicity” OR “secondary malignancy” OR “cognitive impairment” OR “survivorship”).

This is a narrative (expert) review without quantitative meta-analysis. The selection of landmark trials, while systematic in approach, involves qualitative judgment by the authors based on trial prominence, sample size, and relevance to the efficacy–toxicity paradigm. A formal systematic review or PRISMA-ScR-compliant scoping review would require a prospectively registered protocol, a complete flow diagram of identified and screened records, and dual independent reviewer extraction, which were not performed for this manuscript.

Studies were selected based on relevance to pediatric immunotherapy, survivorship outcomes, and the efficacy–toxicity paradigm, prioritizing phase III randomized controlled trials, large cohort studies (*n* > 100), and pivotal phase I/II trials published in English between 2000 and 2026.

## 3. Efficacy Criterion: Maximizing Success at the Initial Stage of Treatment: Breakthroughs in Leukemia Therapy

Acute lymphoblastic leukemia (ALL), the most common childhood cancer, exemplifies both the extraordinary triumphs and the persistent challenges in pediatric oncology. The trajectory of ALL treatment over the past five decades represents one of medicine’s most remarkable success stories. In 1975, a diagnosis of childhood ALL carried a grim prognosis, with fewer than 60% of children achieving long-term survival [10]. Today, that figure stands at approximately 92% in high-income countries, a transformation achieved through successive generations of clinical trials that systematically optimized combination chemotherapy, refined risk stratification, and improved supportive care [11]. Yet, as St. Jude Children’s Research Hospital researcher Rachel Rau observed, “achieving lasting eradication in the remaining patients has proven challenging” [12,13]. This seemingly modest statement belies a profound clinical reality: the final 8–10% of children who still relapse represent thousands of lives lost annually worldwide, and the biological mechanisms that enable leukemic cells to evade even our most intensive therapies continue to resist explanation [14]. Moreover, the 90% who survive often do so at the cost of substantial treatment-related toxicity, raising the question of whether the same survival rates could be achieved with less intensive (and less toxic) approaches in some subgroups [15]. The challenge, then, is twofold: first, to rescue the remaining patients who relapse despite current best therapy, and second, to refine treatment such that the cured population carries a lighter lifelong burden. The emergence of immunotherapies has opened new avenues toward both goals.

A landmark development in the effort to eliminate residual disease came with the addition of blinatumomab to standard chemotherapy regimens for children with standard-risk B-ALL. The phase III Children’s Oncology Group trial AALL1731, presented at the 2024 American Society of Clinical Oncology Annual Meeting, provided the first randomized evidence that incorporating a bispecific T-cell engager (BiTE) into front-line therapy could significantly improve outcomes in a population already considered to have favorable prognosis [16] (Table 1). The trial enrolled over 1440 children with standard-risk B-ALL, randomizing them to receive either conventional chemotherapy alone or chemotherapy with two 28-day cycles of blinatumomab. At three years of follow-up, the results were striking: disease-free survival reached 96.0% for patients receiving blinatumomab plus chemotherapy compared to 87.9% with chemotherapy alone. The reduction in bone marrow relapses, approximately one-third fewer events, was particularly significant. Bone marrow relapse has historically been the most common and most difficult-to-treat pattern of treatment failure in ALL, often requiring intensive salvage chemotherapy, radiation, and hematopoietic stem cell transplantation, with substantial acute and long-term morbidity. By preventing these events, blinatumomab not only improves survival but reduces the need for these toxic salvage interventions [17].

The difference between 87.9% and 96.0% disease-free survival at three years carries tangible human meaning. Among 100 children treated with conventional chemotherapy alone, approximately 12 will experience disease recurrence within three years. Among 100 children receiving the blinatumomab-containing regimen, only 4 will relapse. For every 100 children treated, eight additional families are spared the devastation of relapse, the intensity of salvage therapy, and the uncertainty of subsequent outcomes. Perhaps the most remarkable aspect of the AALL1731 results was the consistency of benefit across all patient subgroups (Table 1). Blinatumomab demonstrated efficacy regardless of risk category, sex, race, ethnicity, or cytogenetic status, effectively neutralizing many of the poor prognostic features that had previously distinguished subsets of standard-risk patients. For example, children with slow early response to induction chemotherapy, traditionally a marker of increased relapse risk, appeared to derive at least as much benefit from blinatumomab as those with rapid response, suggesting that immunotherapy can overcome chemotherapy resistance mechanisms (Table 1).

Blinatumomab’s striking effectiveness stems from how it works, fundamentally different from chemotherapy. While chemotherapy kills all rapidly dividing cells indiscriminately, blinatumomab is a targeted bispecific antibody that links two cell types: cytotoxic T-cells (via CD3) and B-lymphoblasts (via CD19). This connection forms an immune synapse that activates T-cells without needing antigen presentation, essentially turning the patient’s own immune cells against leukemia. Crucially, blinatumomab targets leukemic cells regardless of whether they are dividing, meaning it can eliminate quiescent “sleeping” cells that chemotherapy often misses, including leukemia-initiating cells [18]. It also bypasses common resistance mechanisms like drug efflux pumps. This allows blinatumomab to clear minimal residual disease, the hidden leukemic cells that survive chemo and predict relapse. That is likely why the AALL1731 trial showed such strong clinical benefit.

Blinatumomab exhibits exceptional activity against measurable residual disease (MRD), persistent leukemic cells below the threshold of morphological detection. Unlike chemotherapy, which primarily eliminates cycling cells, blinatumomab activates T-cells against both cycling and quiescent leukemic cells, including leukemia-initiating cells. In the AALL1731 trial, patients achieving MRD-negative status after blinatumomab had 3-year DFS of 98.5% compared to 85.2% for those remaining MRD-positive (HR = 0.21). This MRD-eradicating capacity explains why blinatumomab reduces bone marrow relapse by one-third.

Despite blinatumomab’s efficacy, treatment failure occurs in a subset of patients through two principal mechanisms. First, antigen loss occurs: leukemic cells may downregulate or lose CD19 expression via alternative splicing, truncating mutations, or lineage switch to CD19-negative myeloid phenotype. This mechanism accounts for approximately 10–15% of relapses after blinatumomab. Second, T-cell exhaustion, prolonged or repeated T-cell engagement, upregulates inhibitory receptors (PD-1, TIM-3, LAG-3), reducing cytotoxic capacity. In the AALL1731 trial, patients with pre-existing T-cell exhaustion markers had higher relapse rates. Overcoming these resistance mechanisms requires multi-antigen targeting (e.g., CD19/CD22 bispecific constructs) or checkpoint inhibition.

No discussion of therapeutic efficacy is complete without examining toxicity. In AALL1731, the addition of blinatumomab was associated with increased rates of certain adverse events: sepsis and catheter-related infections occurred in 14.8% of patients receiving the combination versus 5.1% with chemotherapy alone; cytokine release syndrome, generally grade 1–2, was manageable with supportive care; neurologic events including headache, tremor, and rarely seizures were observed; and six deaths in remission occurred among high-risk patients [19]. These toxicities highlight an essential tension in pediatric oncology: improving efficacy often comes at the cost of increased acute toxicity. However, the risk-benefit calculus must also consider the toxicities averted, the salvage therapies, transplant procedures, and late effects that relapse would have necessitated. The net benefit, as measured by both quantity and quality of life, appears to favor the blinatumomab-containing regimen, but ongoing follow-up will be essential to fully characterize the long-term trade-offs.

While blinatumomab represents a breakthrough in front-line therapy, other immunotherapeutic approaches have transformed the landscape for patients who do relapse. Chimeric antigen receptor (CAR) T-cell therapy targeting CD19 has achieved remarkable results in multiply relapsed and refractory B-ALL [20] (Table 1). The ELIANA trial of tisagenlecleucel, the first FDA-approved CAR T-cell product for pediatric ALL, reported an overall remission rate of 81% in children and young adults with relapsed or refractory B-ALL, with 3-year overall survival of 63% and event-free survival of 48% at 3 years [21]. Among patients achieving remission, approximately 60% remained in remission at 3 years without additional therapy. The PLAT-02 trial at Seattle Children’s Hospital evaluated the SCRI-CAR19 CD19-directed CAR T-cell product in children and young adults with relapsed or refractory B-ALL, reporting a 5-year event-free survival of 50%. Notably, some patients achieved continuous remission exceeding 8 years, a remarkable outcome in a population that, before the CAR T-cell era, faced near-zero prospects for long-term survival [22,23]. The extraordinary efficacy of CAR T-cells comes with substantial toxicity: cytokine release syndrome occurred in 77% of patients in ELIANA, with 46% grade 3 or higher; neurotoxicity affected 40% any grade, including life-threatening cerebral edema in rare cases; prolonged B-cell aplasia is universal, requiring immunoglobulin replacement; and infection risk is increased due to B-cell depletion and prior chemotherapy. These toxicities, while manageable in experienced centers, illustrate why CAR T-cell therapy remains reserved for relapsed disease rather than moving to front-line use, at least for now. The challenge for the field is to develop equally effective but less toxic immunotherapies that can be safely deployed earlier in treatment.

**Table 1:** Key Immunotherapy Trials in Pediatric ALL: Comparative Outcomes.

Trial	Phase	Population	Intervention	*N*	Key Efficacy Outcome	Key Toxicity	*p*-Value	Ref.
AALL1731	III	Standard-risk B-ALL, front-line	Chemo + blinatumomab vs. chemo	>1440	3-y DFS: 96% vs. 87.9% (HR 0.39)	Sepsis: 14.8% vs. 5.1%	<0.001	[19]
AALL1331	III	First-relapse B-ALL	Blinatumomab vs. chemo	255	2-y DFS: 54.4% vs. 39.0%	CRS: 15% grade ≥ 3	_	[24]
ELIANA	II	Relapsed/refractory B-ALL	Tisagenlecleucel	75	3-y OS: 63%, 3-y EFS: 48%	CRS: 77% (46% grade ≥ 3)	_	[25]
PLAT-02	I/II	Relapsed/refractory B-ALL	SCRI-CAR19 (CD19-directed CAR T-cell)	45	5-y EFS: 50%	CRS: 88% (23% grade ≥ 3)	0.01	[22]
COG AALL07P1	II	First-relapse B-ALL	Bortezomib + chemotherapy	48	CR2 rate: 80%	Neuropathy: 12%	_	[26]

Another immunotherapeutic strategy involves antibody–drug conjugates, which deliver cytotoxic payloads directly to leukemic cells. Inotuzumab ozogamicin, an anti-CD22 antibody conjugated to calicheamicin, has shown significant activity in relapsed or refractory B-ALL, with complete remission rates of 80% in phase II studies and 70% of responders achieving minimal residual disease-negative status, enabling many patients to proceed to potentially curative hematopoietic stem cell transplantation (Table 2). The primary toxicity concern, hepatic veno-occlusive disease, particularly when followed by transplant, has limited its integration into front-line therapy but demonstrates the potential of targeted toxin delivery.

## 4. The Expanding Role of Immunotherapy

The success of immunotherapy in leukemia has catalyzed investigation across the full spectrum of childhood cancers, revealing that immune-based approaches can achieve meaningful responses even in tumors historically considered resistant to conventional treatment. This expansion of immunotherapy’s reach represents one of the most exciting developments in contemporary pediatric oncology, offering new hope for patients with relapsed or refractory disease while simultaneously raising fundamental questions about optimal timing, sequence, and combination strategies.

Neuroblastoma, an aggressive malignancy of the sympathetic nervous system that accounts for approximately 15% of childhood cancer deaths, has emerged as a particularly compelling example of immunotherapy’s transformative potential [33]. Unlike leukemia, where immunotherapeutic targets like CD19 are uniformly expressed on malignant cells, neuroblastoma required identification of a suitable target, GD2, a disialoganglioside expressed uniformly on neuroblastoma cells but with limited expression on normal tissues, making it an ideal immunotherapeutic target [34] (Table 2). The development of GD2-directed immunotherapies has proceeded through multiple generations, from monoclonal antibodies to chimeric antigen receptor (CAR) T-cells, each building upon lessons learned from predecessors.

The clinical value of GD2-directed immunotherapy for high-risk neuroblastoma has been firmly established, offering complementary insights from different geographic and clinical settings [35,36] (Table 3). The landmark analysis from the Children’s Oncology Group by Desai and colleagues (2022) represents one of the largest cohorts to date, evaluating 1183 patients treated with dinutuximab-based immunotherapy after cessation of random assignment on ANBL0032 [37]. Their findings confirmed previously reported survival outcomes, with 5-year event-free survival (EFS) and overall survival (OS) of 61.1% and 71.9%, respectively, for the entire cohort. Notably, Desai et al. identified important predictive biomarkers, demonstrating that higher peak dinutuximab levels during cycle 1 and a high-affinity FCGR3A genotype were associated with superior EFS. These observations move the field beyond simple efficacy assessment toward personalized immunotherapy strategies.

In the Middle Eastern context, Awwad, Rayis, and Khan (2025) provided much-needed regional data from Saudi Arabia [38]. Their 15-year retrospective single-center study of 41 patients demonstrated that dinutuximab beta therapy significantly improved both OS (46.88 vs. 15.91 months) and EFS (37.42 vs. 14.85 months) compared to a control cohort. The statistical confirmation via log-rank tests (*p* = 0.001 for OS, *p* = 0.007 for EFS) strongly supports the survival benefit of this approach. Importantly, Awwad et al. noted that their findings align with international studies, validating the global applicability of anti-GD2 immunotherapy while addressing a critical gap in Middle Eastern literature.

Complementing these findings, Yu and colleagues (2025) presented the first real-world clinical observational study of dinutuximab beta as first-line maintenance therapy in China [39]. Among 51 newly diagnosed high-risk neuroblastoma patients, they reported impressive 2-year EFS and OS rates of 80.1% and 97.6%, respectively. Notably, Yu et al. demonstrated that patients achieving complete response (CR) before immunotherapy had superior 2-year EFS (94.4%) compared to non-CR patients (72.6%), echoing Desai and colleagues’ finding that end-induction complete or very good partial response predicts better outcomes. Furthermore, Yu et al. introduced an innovative approach combining dinutuximab beta with GM-CSF and VIT chemotherapy for patients with residual disease, showing that this immunochemotherapy regimen was tolerable without requiring treatment discontinuation.

Taken together, these studies converge on several key messages. First, the survival benefits of GD2-directed immunotherapy are reproducible across diverse populations, including North American (Desai et al.), Middle Eastern (Awwad et al.), and Chinese (Yu et al.) cohorts. Second, response status prior to immunotherapy consistently emerges as a prognostic factor. Third, dinutuximab beta appears to offer a favorable safety profile that supports its integration into first-line maintenance protocols, as highlighted by Yu et al. While Desai and colleagues advanced the field by identifying pharmacokinetic and genetic biomarkers, future research should validate these predictors in Asian and Middle Eastern populations where such data remain limited.

This benefit, achieved through antibody-dependent cellular cytotoxicity and complement-dependent cytotoxicity, established that immunotherapy could improve outcomes even in the most aggressive solid tumors and led to FDA approval in 2015 [40]. However, antibody-based approaches require repeated administration, are associated with significant pain due to GD2 expression on peripheral nerves, and show limited efficacy as single agents in bulky relapsed disease.

A unique resistance mechanism in CD19-directed CAR T-cell therapy is lineage switch, transformation of B-ALL into acute myeloid leukemia (AML) or mixed phenotype acute leukemia (MPAL). This occurs in approximately 5–10% of relapsed patients and results from epigenetic reprogramming where leukemic clones retain the original clonal marker (e.g., KMT2A rearrangement) but switch lineage commitment. In the ELIANA trial, lineage switch was associated with extremely poor outcomes (median survival 4.2 months). Detection requires lineage-specific immunophenotyping at relapse, and management necessitates switching to myeloid-directed therapy (Table 3).

A notable finding from this trial was the association between treatment timing and outcomes. Children who received GD2-CART01 after only one or two prior lines of therapy had a five-year survival rate of 89%, compared to 43% for those who had undergone more extensive prior treatment [9,41] (Table 3). This observational comparison, while not randomized and therefore subject to confounding by indication (patients with more aggressive disease may have received more prior lines), generates the strong hypothesis that earlier immunotherapy deployment may improve outcomes. Potential mechanisms include depletion of functional T-cells by multiple chemotherapy lines, selection of more aggressive tumor clones, and induction of an immunosuppressive tumor microenvironment.

The tumor microenvironment (TME) in solid tumors presents unique barriers to CAR T-cell efficacy. Physical barriers include dense extracellular matrix (ECM) composed of collagen, hyaluronan, and proteoglycans that physically impede T-cell infiltration. Chemical barriers include hypoxia (pO2 < 1%) which upregulates HIF-1α and reduces T-cell effector function. Repeated chemotherapy lines worsen these barriers: alkylating agents induce fibroblast activation and ECM remodeling, while platinum compounds promote secretion of immunosuppressive cytokines (TGF-β, IL-10). In the GD2-CART01 trial, patients with ≥3 prior lines had significantly higher TME fibrosis scores on post-relapse biopsy (*p* = 0.008), correlating with poor CAR T-cell penetration. Strategies to overcome TME barriers include ECM-degrading enzymes (hyaluronidase), checkpoint inhibitors, and locoregional delivery (intratumoral or intraventricular injection).

The mechanistic underpinnings of this observation warrant careful consideration. Multiple lines of prior chemotherapy deplete the peripheral T-cell pool, reducing the number and quality of cells available for apheresis and CAR T-cell manufacturing [42]. The T-cells that remain after extensive treatment show evidence of exhaustion, with increased expression of inhibitory receptors like PD-1 and reduced proliferative capacity. The tumor microenvironment in multiply relapsed disease becomes progressively more immunosuppressive, with accumulation of regulatory T-cells, myeloid-derived suppressor cells, and immunosuppressive cytokines [43]. The tumor itself evolves under the selective pressure of multiple therapies, potentially downregulating antigen expression or activating resistance pathways that render it less susceptible to immune attack. Each of these factors likely contributes to the striking difference in outcomes between early and late CAR T-cell administration.

This finding carries a crucial lesson that must inform the future design of clinical trials and treatment algorithms across pediatric oncology: the timing and sequence of novel therapies matter enormously. Historically, new agents have been tested first in the sickest, most heavily pretreated patients, those with relapsed or refractory disease who have exhausted standard options [44]. This approach, driven by ethical considerations and regulatory requirements, made sense when novel therapies offered only incremental benefit and carried unknown risks. However, as immunotherapies demonstrate the potential for transformative benefit, the practice of reserving them for last resort may inadvertently deprive patients of their best chance for a cure. The neuroblastoma data suggest that if GD2-CART01 had been tested only in multiply relapsed patients, its true potential, cure rates approaching 90% when used earlier, might never have been appreciated [45].

The expanding role of immunotherapy is by no means limited to neuroblastoma. Across the spectrum of pediatric solid tumors, promising targets are being identified and translated into clinical candidates. In osteosarcoma, HER2-directed CAR T-cells have shown activity in preclinical models and early-phase trials, with work ongoing to enhance persistence and overcome the immunosuppressive tumor microenvironment [46]. In rhabdomyosarcoma, candidate targets including B7-H3 and EGFR have entered clinical development [47]. In Ewing sarcoma, GD2 is also expressed, raising the possibility that neuroblastoma successes might be replicated [48]. In brain tumors, perhaps the greatest challenge in pediatric oncology, CAR T-cells targeting B7-H3, HER2, and IL13Rα2 are being evaluated in diffuse intrinsic pontine glioma and other high-grade gliomas, with early signals of radiographic responses in tumors that have historically been uniformly fatal [49].

The challenge of the immunosuppressive tumor microenvironment looms particularly large in solid tumors and represents a key barrier to immunotherapy efficacy. Unlike leukemia, where malignant cells circulate in blood and bone marrow accessible to immune effectors, solid tumors erect physical and biochemical barriers to immune infiltration. They recruit regulatory T-cells and myeloid-derived suppressor cells, secrete immunosuppressive cytokines like TGF-β and IL-10, upregulate checkpoint molecules that induce T-cell exhaustion, and establish hypoxic and acidic microenvironments that impair immune function [50]. Overcoming these barriers will require combination strategies that pair CAR T-cells with checkpoint inhibitors, oncolytic viruses that inflame the tumor microenvironment, or agents that deplete immunosuppressive populations.

**Table 3:** Improving Survival in Childhood Malignancies: Key Lessons and Future Directions.

Domain	Key Findings & Data	Core Lessons	Ref.
The Central Challenge	80% survival (HICs)	Cure ≠ health; success requires balancing efficacy with quality of survival	[51]
	95% of survivors have ≥1 late effect; 60–90% carry chronic conditions		
Immunotherapy: Leukemia	AALL1731 (Blinatumomab): 3-y DFS 96% vs. 87.9%; sepsis 14.8% vs. 5.1%	Immunotherapy improves outcomes even in favorable groups; toxicity limits front-line use	[52]
	ELIANA/PLAT-02 (CAR-T): 80% remission; 3–5 y EFS 48–50% in R/R disease; CRS in 77–88%		
Immunotherapy: Solid Tumors	ANBL0032 (Dinutuximab): 5-y EFS 63% vs. 46%	Solid tumors are immunoresponsive; timing is critical—earlier deployment yields dramatically better outcomes	[53]
	GD2-CART01 (CAR-T): 5-y OS 89% (early use) vs. 43% (late use)		
The Relapse Challenge	Phase 1 trial outcomes (R/R disease): Median OS 13.1 months	Relapse is the leading cause of death; salvage therapy for solid tumors remains inadequate	[54]
	Response rates: Hematologic (53%), Brain (21%), Solid tumors (16%)		
	Relapse drives clonal evolution and resistance		
Toxicity Burden	Acute: Treatment-related mortality; infections 3 × higher with intensification	Toxicity affects nearly all survivors across physical, psychological, and economic domains; survivorship care is essential	[55]
	Long-term: 60–90% chronic conditions (cardiac, second cancers, infertility, cognitive)		
	Psychosocial: Anxiety 20–35%, depression 15–25%, suicidal ideation 2–3 × higher		
	Economic: Lifetime excess cost > $50,000/survivor		
Risk-Adapted Strategies	Medulloblastoma molecular stratification: Identifies ~40% of patients eligible for therapy reduction (e.g., WNT subgroup)	Matching intensity to biological risk preserves efficacy while reducing toxicity	[56]
Critical Gaps & Future Directions	Surveillance: Optimal screening intervals unknown	Need for: prospective studies, validated surrogates, comparative effectiveness research, and globally scalable solutions	[57]
	Genetics: Predictors of toxicity incomplete		
	Psychosocial: Interventions lacking		
	Mechanisms: Link between childhood therapy and decades-later effects poorly understood		
	Global: HIC survival 80% vs. LMIC < 30%		

### Molecular Risk Stratification: The Medulloblastoma Paradigm

Medulloblastoma, the most common malignant brain tumor in children, exemplifies how molecular characterization can enable treatment de-escalation without compromising efficacy. Historically, all children with medulloblastoma received craniospinal irradiation and intensive chemotherapy, resulting in substantial neurocognitive toxicity, endocrine dysfunction, hearing loss, and secondary malignancies, particularly devastating in young children whose developing brains are highly vulnerable to radiation-induced injury [58]. The discovery of four distinct molecular subgroups, WNT, SHH, Group 3, and Group 4, has fundamentally transformed this landscape [59].

Patients with WNT-subgroup medulloblastoma, accounting for approximately 10–15% of cases, harbor characteristic CTNNB1 mutations and are typically older children with classic histology. These tumors have an excellent prognosis, with 5-year survival exceeding 90% on standard therapy, driven in part by their enhanced sensitivity to radiation and chemotherapy due to defective DNA repair mechanisms and increased blood–brain barrier permeability. This observation led to prospective de-escalation trials. The St. Jude Medulloblastoma protocol (SJMB12) demonstrated that reducing craniospinal radiation dose from 23.4 Gy to 18.0 Gy in WNT-patients achieved 5-year progression-free survival of 95% with significantly reduced cognitive decline, as measured by processing speed and working memory indices [60]. Similarly, the German HIT-SIOP PNET5 trial evaluated complete avoidance of radiation in young children with WNT tumors after intensive chemotherapy alone, with early results suggesting feasibility in carefully selected patients [61].

Beyond the WNT subgroup, molecular stratification has further refined risk assessment. SHH-subgroup tumors, which harbor PTCH1, SMO, or SUFU mutations, have variable outcomes depending on additional clinical factors and specific genetic alterations. Patients with TP53-mutant SHH tumors carry a very poor prognosis and are not candidates for de-escalation, while SHH in infants without high-risk features may be managed with reduced-intensity approaches [62]. Group 3 and Group 4 tumors, now further subdivided by the presence of MYC amplification, isochromosome 17q, and transcriptional subtyping, have intermediate to poor outcomes. Recent studies have demonstrated that low-risk Group 4 patients, characterized by the absence of MYC amplification and specific chromosomal gains, may also benefit from treatment de-escalation [63,64].

Collectively, molecular stratification identifies approximately 40–50% of medulloblastoma patients (WNT and low-risk SHH/Group 4) as eligible for therapy reduction, preserving excellent efficacy while substantially reducing long-term toxicity including hearing loss, endocrine dysfunction (growth hormone deficiency, hypothyroidism, gonadal failure), and neurocognitive impairment. Long-term follow-up studies confirm that survivors treated on de-escalated protocols have better quality of life, academic achievement, and employment outcomes compared to those receiving standard-dose craniospinal radiation [56].

This paradigm, matching treatment intensity to biological risk, serves as a powerful blueprint for other pediatric malignancies. The success of molecular stratification in medulloblastoma has inspired similar approaches in ependymoma, rhabdomyosarcoma, and neuroblastoma, where genomic biomarkers are increasingly guiding treatment intensity. As next-generation sequencing and methylation profiling become standard diagnostic tools, the integration of molecular risk stratification into upfront treatment planning promises to further improve the therapeutic index, maximizing cure while minimizing lifelong treatment-related morbidity.

## 5. The Problem of Relapse: Lessons from Unsuccessful Treatment

**Figure 1:**
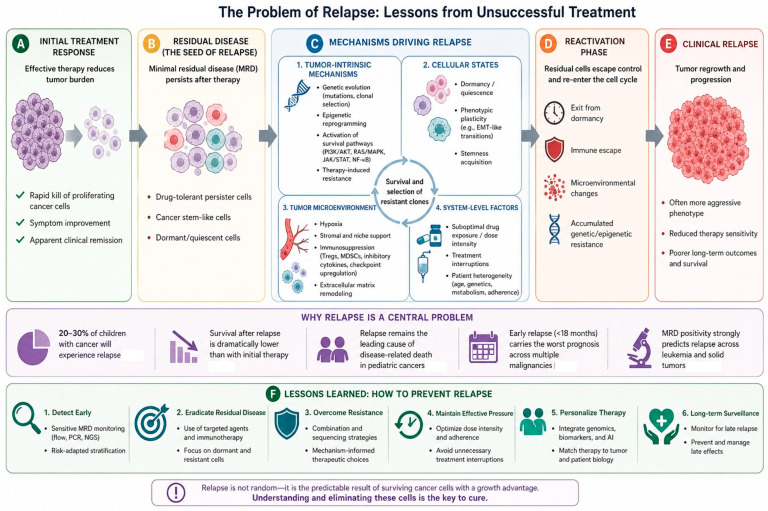
The problem of relapse: lessons from unsuccessful treatment. This figure summarizes the progression from initial treatment response to cancer relapse. (**A**) Therapy reduces tumor burden and induces remission. (**B**) Minimal residual disease (MRD) persists, including drug-tolerant, stem-like, and dormant cells. (**C**) Relapse is driven by tumor-intrinsic changes (genetic/epigenetic), cellular plasticity, microenvironmental support, and system-level factors, leading to selection of resistant clones. (**D**) Residual cells re-activate through escape from dormancy and immune control. (**E**) Clinical relapse follows, often with more aggressive and therapy-resistant disease. (**F**) Lessons learned for relapse prevention include early MRD detection and risk stratification, eradication of residual disease using targeted and immunotherapeutic approaches, overcoming resistance through mechanism-informed combination therapies, maintaining effective treatment pressure and adherence, personalizing therapy using genomic and biomarker-guided strategies, and implementing long-term surveillance to detect late relapse and treatment-related effects. AI tool ChatGPT-4 (OpenAI, San Francisco, CA, USA) was employed for the generation of Figure 1.

Despite five decades of steady progress in front-line therapy, relapse remains the leading cause of death in childhood cancer, a sobering reality that demands continued attention to understanding why initial treatment fails and how salvage outcomes can be improved. For the approximately 20% of children with cancer who will experience disease recurrence, the prognosis is often devastating, and the therapeutic options limited [6]. Understanding the patterns, predictors, and biology of relapse provides critical insights that can inform both front-line treatment intensification strategies and the development of more effective salvage approaches (Figure 1).

Contemporary data from early-phase clinical trials paint a stark picture of what happens when initial treatment fails. A comprehensive analysis of 224 pediatric patients enrolled in phase 1 trials at a major cancer center revealed median overall survival of only 13.1 months following trial enrollment [65]. This figure, while expected in a population with multiply relapsed or refractory disease, nonetheless carries profound implications: even with access to the newest experimental agents, most children with relapsed cancer will not survive beyond one year. Although 27.6% of patients achieved an objective response to trial therapy, these responses were often short-lived, with median treatment duration of just 1.5 months [66]. This pattern, initial responses that rapidly extinguish, reflects the aggressive biology of relapsed disease, where tumors have already demonstrated their ability to evade multiple lines of conventional therapy and often harbor complex resistance mechanisms.

Rami et al. (2025) analyzed 224 pediatric patients enrolled in phase 1 trials at Dana-Farber/Boston Children’s Hospital (2011–2019) [65]. Median overall survival from trial enrollment was 13.1 months. Overall response rate was 27.6%, but varied significantly by disease type: hematologic malignancies responded best at 52.9%, followed by brain tumors (20.5%) and solid tumors (15.8%). The high response rate in blood cancers reflects both their inherent chemosensitivity and the success of targeted immunotherapies like blinatumomab and CAR T-cells. Following trial participation, 62.5% of patients received additional therapy. Brain tumors showed intermediate response rates of 20.5%, a figure that, while modest, represents progress in tumors where few effective options exist [65]. Most concerning were solid tumors, where response rates reached only 15.8%, meaning that more than eight of every ten children with relapsed solid tumors derived no objective benefit from experimental therapy. These disparities reflect fundamental biological differences: hematologic malignancies circulate in blood and bone marrow, accessible to immune effectors and systemically administered therapies, while solid tumors establish immunosuppressive microenvironments, develop physical barriers to drug penetration, and evolve complex resistance mechanisms through genomic instability.

The persistently low 15.8% response rate in pediatric phase 1 solid tumor trials (Rami et al., 2025) reflects three interrelated factors: targeting inappropriate pathways, inadequate drug penetration, and the fundamentally distinct biology of relapsed disease [65]. Genomic evidence from the SMPaeds study (George et al., 2024) demonstrates that relapsed tumors are not simply recurrences of the original disease [67]. Profiling 401 patients revealed significant enrichment of mutations in epigenetic modifier genes (ATRX, SETD2, ARID1B, CREBBP) at relapse, indicating positive selection for resistant clones with distinct epigenetic profiles. If phase 1 agents target pathways relevant at diagnosis rather than evolutionarily selected relapse drivers, low response rates are predictable. Cell-free DNA analysis further showed that relapsed tumors harbor greater intratumoral heterogeneity, suggesting subclonal populations escape initial therapy and seed recurrence.

Drug penetration barriers compound this problem. Patel et al. (2021) note that intravenously administered agents face multiple obstacles [68]. It includes, but not limited to, plasma protein binding reduces free drug fractions; non-specific uptake by healthy organs depletes circulating concentrations; the blood–brain barrier limits central nervous system penetration; and heterogeneous tumor vascularization with elevated interstitial pressure prevents uniform distribution. The authors emphasize that poor understanding of these barriers, combined with limited pediatric pharmacokinetic data, prevents existing therapies from achieving maximal efficacy.

Most concerning is evidence that relapsed solid tumor biology may be fundamentally more challenging than appreciated. A systematic review by Cohen et al. (2020) analyzed 109 pediatric Phase I oncology trials conducted between 2012 and 2017, including a total of 2713 patients [69]. The study evaluated the safety and early efficacy of investigational therapies in children with cancer. Dose-limiting toxicity occurred in approximately 12.1% of patients, indicating an acceptable safety profile for early-phase trials. Among 2143 patients evaluable for treatment response, the pooled objective response rate was 15.3%, while 39% of trials reported no objective responses. Trials involving targeted therapies demonstrated lower toxicity compared to cytotoxic treatments, with similar response rates. The findings suggest that although pediatric Phase I trials are generally safe; their overall therapeutic benefit remains modest.

The biology of relapse provides clues to why salvage therapy so often fails. Relapsed tumors are not simply a repetition of the original disease but have evolved under the selective pressure of initial therapy. They acquire new mutations, activate alternative signaling pathways, and remodel their microenvironments to resist further treatment. In leukemia, relapse clones often show enrichment for mutations in genes regulating chromatin modification, suggesting that epigenetic dysregulation contributes to treatment resistance. In neuroblastoma, relapsed tumors frequently amplify MYCN or activate RAS-MAPK pathway mutations not present at diagnosis [70]. In medulloblastoma, relapse may involve switching between molecular subgroups, with tumors that were originally Sonic Hedgehog-driven recurring as Group 3 or Group 4 [71]. This clonal evolution means that the tumor being treated at relapse is biologically distinct from the original disease, and therapies selected based on diagnostic characteristics may be mismatched to the actual target.

Circulating tumor DNA (ctDNA) analysis has emerged as a powerful tool for relapse monitoring. Serial ctDNA sequencing can detect clonal evolution before clinical relapse, identifying emerging mutations in epigenetic modifiers (ATRX, SETD2, ARID1B) that confer therapy resistance. In the SMPaeds study, ctDNA detected relapse a median of 4.2 months before radiographic or clinical evidence (range 1.2–9.5 months). For patients with neuroblastoma, ctDNA detection of MYCN amplification or RAS-MAPK mutations predicted non-response to salvage therapy with 88% sensitivity. Incorporating ctDNA into surveillance protocols enables earlier intervention, potentially before tumors establish full resistance.

The timing of relapse provides additional prognostic information and may reflect underlying biology. Early relapses, those occurring within 18 months of diagnosis, generally carry worse prognosis than late relapses, likely reflecting inherent chemoresistance of the original disease. In ALL, early bone marrow relapse has historically been associated with survival rates below 20%, while late relapse occurring more than three years from diagnosis may be salvageable in 40–50% of patients [72]. In neuroblastoma, patients who relapse during or immediately after front-line therapy have near-zero long-term survival, while those with late, isolated relapses may achieve durable second remissions. These patterns inform clinical decision-making, with early relapse generally prompting consideration of novel agents and transplant. In contrast, late relapse may be approached with reinduction therapy similar to front-line regimens.

Analysis of relapse patterns also informs front-line treatment strategies by revealing weaknesses in current approaches that allow residual disease to persist and eventually recur. The blinatumomab trial AALL1731 provided a striking example of this principle: while the addition of blinatumomab to chemotherapy reduced bone marrow relapses by approximately one-third, central nervous system relapses remained relatively unchanged. This observation reflects the drug’s limited penetration into the CNS, where the blood–brain barrier excludes large antibody constructs like blinatumomab. The implication is clear: even the most effective systemic immunotherapy cannot address leukemic cells sequestered in sanctuary sites. Effective front-line therapy must therefore combine modalities that reach all compartments where disease may hide, systemic chemotherapy, immunotherapy, and CNS-directed therapy including intrathecal chemotherapy and, in some cases, cranial radiation.

The psychosocial dimensions of relapse deserve explicit attention. For families, relapse represents a devastating reversal of fortune, shattering the hope that treatment had been successful and requiring reengagement with the medical system at a time of emotional exhaustion. Children who relapse face not only the physical challenges of salvage therapy but also the psychological burden of confronting mortality at a young age. Siblings, who may have already experienced disruption during initial treatment, must again navigate family stress and uncertainty. Healthcare providers, who develop deep relationships with patients and families over months or years of treatment, experience moral distress when relapses occur despite their best efforts. Comprehensive care for relapsed patients must address these psychosocial dimensions alongside medical management, integrating palliative care principles even when pursuing aggressive salvage therapy.

The economic burden of relapse is substantial and often underappreciated. Families face additional months or years of treatment-related expenses, lost wages, and travel costs for specialized care that may be available only at distant centers. Health systems bear the cost of intensive salvage regimens, prolonged hospitalizations, and, ultimately, end-of-life care for patients who cannot be salvaged. These economic considerations, while secondary to clinical imperatives, must inform discussions about resource allocation and the value of relapse prevention strategies.

## 6. The Toxicity Burden: The Hidden Determinant of Survival

### 6.1. Short-Term Complications of Treatment: An Immediate Threat to the Success of Treatment

The immediate toxicities of cancer therapy represent a constant, ever-present threat to treatment success. These acute complications can derail planned therapy, necessitate dose reductions that may compromise efficacy, and in the worst cases, prove fatal even when the cancer itself is controlled. In the era of increasingly intensive treatment regimens, understanding and managing these short-term toxicities has become essential to achieving the survival gains those modern protocols promise.

The blinatumomab trial AALL1731 provides an instructive example of the toxicity trade-offs inherent in therapeutic intensification [73] (Table 4). While the addition of this bispecific T-cell engager to conventional chemotherapy significantly improved disease-free survival, it came with increased rates of certain adverse events. Sepsis and catheter-related infections occurred in 14.8% of patients receiving the combination versus 5.1% with chemotherapy alone, a nearly threefold increase in serious infectious complications [16]. Six deaths in remission occurred among high-risk patients, underscoring that treatment-related mortality remains a real and present danger even as we celebrate improvements in disease control. These deaths represent the ultimate paradox of modern oncology: children dying from the treatment intended to save them.

The mechanisms underlying this increased infectious risk are multifaceted. Blinatumomab’s mechanism of action involves activating T-cells to kill leukemic cells, but this immune activation can also impair normal immune function [74]. The drug causes transient B-cell depletion, reducing antibody production and increasing susceptibility to infection. The chemotherapy backbone itself causes prolonged neutropenia, compromising the innate immune system. Central venous catheters, essential for delivering prolonged infusions, provide portals of entry for bacteria. Together, these factors create a perfect storm of infectious risk that requires aggressive supportive care, including prophylactic antibiotics, antifungal agents, and intravenous immunoglobulin in some patients.

Beyond infectious complications, blinatumomab is associated with unique toxicities related to its immune-activating mechanism [75]. Cytokine release syndrome, characterized by fever, hypotension, and respiratory insufficiency, results from massive T-cell activation and cytokine release. While generally mild to moderate in the AALL1731 trial, cytokine release syndrome can be life-threatening and requires careful monitoring and management with tocilizumab or corticosteroids. Neurologic events, including headache, tremor, somnolence, and rarely seizures, reflect the drug’s effects on the central nervous system and may be related to T-cell trafficking into the brain. These neurologic toxicities are generally reversible but can be distressing for patients and families and may require temporary treatment interruption.

Early-phase trials provide a broader window into the full spectrum of treatment toxicity encountered when pursuing new therapeutic options [65] (Table 4). The pattern of toxicities in phase 1 trials varies substantially by drug class and mechanism of action. Conventional cytotoxic agents cause predictable myelosuppression, mucositis, and organ toxicity related to their effects on rapidly dividing normal tissues. Targeted therapies produce more mechanism-based toxicities: kinase inhibitors may cause hypertension, cardiac dysfunction, or metabolic abnormalities related to their effects on normal signaling pathways. Immunotherapies produce inflammatory toxicities from immune activation, including cytokine release syndrome, immune effector cell-associated neurotoxicity syndrome, and autoimmune phenomena affecting virtually any organ system. Understanding these toxicity patterns is essential for anticipating complications, implementing preventive strategies, and managing adverse events when they occur.

### 6.2. Long-Term Consequences: Survival Lessons

Perhaps the most profound lessons about toxicity emerge not from active treatment but from decades of survivorship research that has followed cured patients into adulthood. As survival rates have improved and the first generations of childhood cancer survivors have aged into their fifth, sixth, and seventh decades, the long-term consequences of curative therapy have come into sharp focus.

The statistics are sobering and demand attention from everyone involved in pediatric oncology. Approximately 95% of childhood cancer survivors will experience at least one health-related issue related to their prior treatment during their lifetime, with perhaps one-third experiencing a severe or life-threatening late effect [76] (Table 4). Studies of large survivorship cohorts have indicated that 60 to 90% of adult survivors of childhood cancer have at least one chronic health condition, and many have multiple coexisting conditions [77,78]. This means that for every ten children cured of cancer, six to nine will carry a lifelong burden of treatment-related morbidity that affects their health, quality of life, and functional status.

Among the most heavily affected subgroups are survivors of pediatric leukemia and neuroblastoma. For survivors of childhood acute myeloid leukemia (AML), the cumulative burden of chronic health conditions is starkly elevated compared to the general population. A report from the St. Jude Lifetime Cohort Study by Bhatt and colleagues demonstrated that by age 40 years, survivors treated with hematopoietic cell transplantation (HCT) had an average of 17.4 grade 1–4 chronic conditions, while those treated with conventional therapy averaged 9.3 conditions, compared to only 3.8 conditions in community controls [79]. Specific late effects included cardiomyopathy (11.9% in conventionally treated survivors), primary hypogonadism, and thyroid dysfunction, as well as neurocognitive impairments across multiple domains. Similarly, survivors of childhood acute lymphoblastic leukemia (ALL) face risks of anthracycline-induced cardiomyopathy, cognitive dysfunction, obesity, and osteopenia at young ages [80,81].

For neuroblastoma survivors, the late effect burden is equally concerning. A population-based cohort study from the Adult Life after Childhood Cancer in Scandinavia (ALiCCS) study by Norsker and colleagues found that 5-year survivors of neuroblastoma had a significantly increased risk of hospitalization for somatic late effects, with standardized hospitalization rate ratios of 3.6 for endocrine diseases, 3.1 for circulatory diseases, and 3.0 for nervous system diseases [82]. More recently, Henderson and colleagues (2025) published the first study examining late effects in high-risk neuroblastoma survivors treated with modern therapies, including multiple stem cell transplants and immunotherapy. Among 375 survivors, they reported a striking 72% prevalence of moderate-to-severe hearing loss, 51% being underweight, and 24% with growth failure. Importantly, most participants had at least two clinically important late effects, and longer follow-up was associated with higher prevalence [83].

The variability in late effect severity among similarly treated survivors has increasingly focused attention on genetic susceptibility. A systematic review by Bolier and colleagues (2025) examined the evidence for genetic variants associated with late effects including hearing impairment, metabolic syndrome, and gonadal insufficiency [84]. While 85 variants were significantly associated in individual studies, substantial uncertainty remains, and only one variant (rs4646316/COMT) remained significant in meta-analysis [84]. The authors emphasized the need for international collaboration and methodological harmonization to bridge the gap between research and clinical practice.

The spectrum of late effects is remarkably broad, reflecting the diverse therapies used and organ systems affected (Table 4). Cognitive impairment affects survivors of central nervous system-directed therapy, including cranial radiation and intrathecal chemotherapy, manifesting as deficits in attention, processing speed, memory, and executive function that can impact educational attainment, employment, and independent living [85]. Fertility problems result from gonadal toxicity of alkylating agents and radiation, affecting the ability of survivors to build families of their own and causing psychological distress related to lost reproductive potential. Growth abnormalities, including short stature and growth hormone deficiency, affect survivors who received cranial or total body irradiation, requiring hormone replacement and sometimes surgical interventions. Organ system damage can affect virtually any organ: cardiomyopathy from anthracyclines, pulmonary fibrosis from bleomycin or radiation, nephropathy from cisplatin or ifosfamide, hepatopathy from methotrexate or radiation, and endocrinopathies affecting thyroid, adrenal, and gonadal function. Secondary malignancies represent perhaps the most feared late effect, with survivors facing substantially increased risks of therapy-related leukemias, solid tumors in radiation fields, and cancers associated with genetic predisposition syndromes.

The cumulative burden of survivorship affects not only physical health but psychological well-being in ways that are increasingly recognized as central to quality of life. Recent data have highlighted an almost occult prevalence of anxiety, depression, somatic distress, and suicidal ideation among survivors, psychological sequelae that often go unrecognized and untreated in medical follow-up focused on physical health. The prevalence of clinically significant anxiety approaches 20–35% in some studies, compared to 10–15% in sibling controls [86]. Depression affects 15–25% of survivors, with rates highest among those with significant physical late effects, chronic pain, or visible sequelae of treatment. Post-traumatic stress symptoms, including intrusive thoughts about cancer, avoidance of medical care, and hyperarousal, affect a substantial minority of survivors and their family members. Suicidal ideation is 2–3 times more common among survivors than the general population, particularly in the context of uncontrolled pain, functional impairment, or social isolation.

The economic burden of late effects is substantial and often borne by survivors and their families. Direct medical costs include ongoing surveillance, management of chronic conditions, treatment of secondary malignancies, and medications for hormone replacement, cardiac function, and other sequelae. Indirect costs include lost productivity from disability, reduced employment, and premature mortality. Studies have estimated that the lifetime excess cost of care for a childhood cancer survivor exceeds $50,000, with substantial variation based on treatment exposures and resulting late effects. For health systems, the cumulative burden of caring for a growing population of survivors, expected to exceed 500,000 in the United States alone by 2030, represents a significant and growing financial obligation [87].

The recognition of this long-term toxicity burden has driven fundamental changes in how we think about treatment success. The traditional metric of five-year survival, while still important, is increasingly recognized as inadequate for capturing the full impact of cancer therapy. Instead, survivorship research has introduced concepts of “survival with minimal morbidity,” “quality-adjusted life years,” and “patient-reported outcomes” that attempt to quantify not just quantity but quality of survival. These metrics are increasingly incorporated into clinical trials as secondary endpoints and are beginning to influence treatment recommendations and regulatory approvals.

### 6.3. Strategies for Toxicity Mitigation

Effective toxicity mitigation integrates four complementary approaches: pharmacogenomic-guided dosing, AI-based risk prediction, pharmacologic cardioprotection, and structured survivorship care.

First, pharmacogenomic-guided dosing helps individualize therapy based on inherited drug metabolism. Variants in *TPMT* and *NUDT15* strongly predict severe myelosuppression from thiopurines, which are mainstays of ALL maintenance therapy. According to CPIC guidelines, intermediate metabolizers require 50–80% dose reduction, while poor metabolizers need alternative agents. Implementing this approach reduces severe neutropenia from 35% to less than 10% without compromising disease-free survival.

Second, AI-based toxicity prediction offers a powerful tool for pre-treatment risk stratification. Machine learning models that integrate treatment parameters (cumulative anthracycline dose, radiation fields), demographic factors (age at treatment, sex), and genetic variants (*RARG*, *SLC28A3*) can predict anthracycline-induced cardiotoxicity with AUC values of 0.85–0.92. Although prospective validation is ongoing, such models may soon guide treatment de-escalation or early cardioprotective interventions in high-risk patients.

Third, pharmacologic cardioprotection provides a direct strategy to mitigate the most common and serious late effect. Dexrazoxane, an iron chelator, reduces anthracycline-induced oxidative stress by binding free iron and preventing formation of toxic reactive oxygen species. Meta-analysis demonstrates a 65% relative reduction in cardiac events (RR = 0.35, 95% CI: 0.22–0.56) without evidence of compromised anti-tumor efficacy. ASCO currently recommends dexrazoxane for children receiving cumulative anthracycline doses of 300 mg/m^2^ or higher.

Finally, structured survivorship care models ensure that late effects are detected early and managed effectively. Risk-stratified follow-up per COG guidelines includes annual echocardiography for anthracycline-exposed survivors (≥250 mg/m^2^) and low-dose mammography combined with breast MRI for chest radiation-exposed females (≥15 Gy) starting at age 25. Critically, structured transition programs from pediatric to adult survivorship care improve follow-up adherence by approximately 40%, bridging a major gap in long-term care. Taken together, these strategies shift toxicity management from reactive to proactive, offering the potential to preserve both cure rates and quality of life for childhood cancer survivors.

## 7. Knowledge Gaps and Future Directions

Despite five decades of transformative progress in childhood cancer treatment, significant knowledge gaps remain in our understanding of how to optimize the delicate balance between efficacy and toxicity. These gaps span the entire continuum of care, from initial diagnosis through active treatment and into the decades of survivorship that follow. Identifying and addressing these gaps is essential to ensuring that the next generation of children with cancer achieves not only higher cure rates but also healthier, fuller lives beyond their disease.

The most pressing knowledge gaps can be organized across several domains. In terms of long-term surveillance, there are limited data on optimal strategies for detecting late effects. While consensus-based guidelines recommend specific screening modalities and frequencies based on treatment exposures, these recommendations are often derived from expert opinion rather than rigorous evidence. For example, echocardiography is recommended every one to five years for anthracycline-exposed survivors, but the optimal interval remains unknown. More frequent screening might detect cardiotoxicity earlier but increases healthcare costs and patient burden, while less frequent screening risks missing the window for intervention. Similarly, mammography is recommended for chest radiation-exposed females starting at age 25 or eight-years post-radiation, but the sensitivity and specificity of mammography in this population with dense breasts and radiation-induced changes has not been systematically evaluated. The comparative effectiveness of different surveillance strategies, their ability to detect late effects early, improve outcomes, and do so cost-effectively, remains largely unknown and requires prospective study.

Our understanding of genetic predispositions to treatment toxicity remains incomplete. While pharmacogenomic discoveries have identified variants in genes like *TPMT*, *NUDT15*, and *GSTA1* that predict toxicity from specific chemotherapeutic agents, these represent only a fraction of the heritable factors that influence treatment complications. Genome-wide association studies have begun to identify additional loci associated with anthracycline cardiotoxicity, cisplatin ototoxicity, and radiation-induced second malignancies, but these findings require validation and translation into clinical practice. The interaction between genetic susceptibility and other factors, age at treatment, sex, race, ethnicity, concurrent medications, and health behaviors, adds further complexity that remains poorly understood. Prospective studies incorporating comprehensive germline genotyping with detailed toxicity phenotyping are needed to build predictive models that can identify high-risk patients before treatment begins and guide preventive strategies.

Research on psychosocial and cognitive late effects has lagged behind investigation of physical sequelae, despite the profound impact these issues have on survivors’ quality of life. While we know that survivors face increased risks of anxiety, depression, and neurocognitive impairment, we have limited understanding of the mechanisms underlying these effects, the optimal timing and modalities for screening, and the effectiveness of interventions. For cognitive impairment following cranial radiation, we do not know whether cognitive rehabilitation programs, pharmacologic interventions, or educational accommodations can mitigate deficits, nor do we understand the windows of neuroplasticity during which interventions might be most effective. For psychological distress, we lack evidence-based guidelines for screening frequency, validated instruments for different age groups, and proven interventions tailored to survivors’ unique needs. The interaction between physical and psychological late effects, for example, how chronic pain or visible disfigurement contributes to depression and anxiety, remains underexplored and underappreciated.

The economic dimensions of survivorship care remain underexplored. While we know that survivors face substantial direct and indirect costs, we have a limited understanding of the cost-effectiveness of different surveillance strategies, the economic impact of late effects on survivors and families, and the optimal models of care delivery from a health systems perspective. As the survivor population grows to exceed 500,000 in the United States alone by 2030, these economic considerations will become increasingly important for resource allocation and healthcare policy. Comparative effectiveness research that evaluates both clinical outcomes and costs is needed to inform decisions about which surveillance strategies to implement, which interventions to reimburse, and how to structure survivorship care programs for maximum efficiency and impact.

The biological mechanisms linking childhood cancer therapy to late effects decades later represent another frontier requiring investigation. While we understand that anthracyclines cause acute cardiotoxicity through oxidative stress, we do not fully understand why cardiac dysfunction progresses over decades, why some survivors develop heart failure while others with similar exposures do not, or how aging interacts with treatment-induced damage to produce clinical disease. For second malignancies, we know that radiation causes DNA damage that can lead to cancer, but we cannot predict which radiation-exposed survivors will develop secondary tumors, nor do we understand the role of genetic susceptibility, environmental factors, or health behaviors in modifying risk. Elucidating these mechanisms could identify targets for intervention, agents that could be administered years after treatment to slow or prevent the progression of late effects, much as statins are used to prevent cardiovascular disease in at-risk populations.

The development of interventions to prevent or mitigate late effects has progressed slowly, in part because of the long latency between treatment and clinical manifestations. Clinical trials testing cardioprotective agents, cognitive rehabilitation programs, or psychosocial interventions require decades of follow-up and large sample sizes, making them expensive and difficult to conduct. Surrogate endpoints that predict long-term outcomes, subclinical cardiac dysfunction on echocardiography, cognitive deficits on neuropsychological testing, inflammatory biomarkers, could accelerate research by enabling shorter-term trials, but these surrogates require validation. Adaptive trial designs, platform trials, and master protocols that can efficiently test multiple interventions in survivorship populations represent promising methodological innovations that could accelerate progress.

Innovative clinical trial designs can substantially accelerate progress in oncology and beyond by aligning patient selection with tumor or host biology. Basket trials enroll patients with different tumor types sharing a common molecular alteration (e.g., NTRK fusions, BRAF V600E mutations) to test a single targeted therapy, allowing signal detection across rare cancers [88,89]. Umbrella trials assign patients with a single disease, such as relapsed acute lymphoblastic leukemia (ALL), to multiple targeted arms based on molecular profiling, maximizing information from a heterogeneous patient population [90,91]. The NCI-MATCH pediatric framework exemplifies these designs in practice: across 388 evaluable patients, 12% of those with refractory cancers were successfully matched to a targeted therapy, with an overall response rate of 25%, including durable responses in rare tumor types [92,93]. Applying these adaptive frameworks to toxicity mitigation is a logical next step. For example, an umbrella trial in patients receiving cardiotoxic chemotherapy (e.g., anthracyclines) could randomize high-risk individuals, identified by genetic profiles (e.g., *RARG*, *UGT1A6*, *SLC28A3* variants), to different cardioprotective strategies (e.g., dexrazoxane, beta-blockers, or statins) [94,95]. Such a design would generate prospective, high-level evidence efficiently, enabling personalized supportive care and reducing the burden of treatment-related morbidity.

## 8. Conclusions

The preceding analysis, based on a narrative synthesis of landmark trials, supports several clinically relevant observations rather than graded recommendations. First, evidence from the randomized phase III AALL1731 trial indicates that adding blinatumomab to front-line chemotherapy for standard-risk B-ALL improves 3-year disease-free survival from 87.9% to 96.0% (HR = 0.39), although this benefit is accompanied by increased sepsis risk (14.8% vs. 5.1%). Second, for relapsed or refractory B-ALL, CD19-directed CAR T-cell therapy (tisagenlecleucel) achieves remission in approximately 80% of patients, with 48% 3-year event-free survival, as demonstrated in the ELIANA trial. Third, observational data from the GD2-CART01 trial suggest that earlier use (after 1–2 prior lines) is associated with superior 5-year survival (89% vs. 43% for later use), though this finding requires prospective confirmation due to potential confounding by indication. Fourth, pharmacogenomic guidance (*TPMT*/*NUDT15* genotyping for thiopurine dosing) and pharmacologic cardioprotection (dexrazoxane for cumulative anthracycline doses ≥300 mg/m^2^) represent evidence-supported strategies to mitigate treatment-related toxicity. Fifth, structured survivorship surveillance per Children’s Oncology Group guidelines—including annual echocardiography for anthracycline-exposed survivors (≥250 mg/m^2^), breast MRI with mammography starting at age 25 for chest radiation-exposed females (≥15 Gy), and structured transition to adult care—improves follow-up adherence. In summary, achieving a cure with quality in pediatric oncology requires moving from general principles to specific, evidence-informed actions. However, given the narrative nature of this review, these observations should be interpreted as hypothesis-generating and clinically informative rather than as formal GRADE recommendations.

## Figures and Tables

**Table 2:** Mechanisms of Resistance and Immunotherapy Targeting.

Resistance Mechanism	Description	Molecular Pathway	Immunotherapy Strategy	Clinical Example	Ref.
Antigen downregulation	Trogocytosis (transfer of CD19 from target cell to CAR T-cell surface) or alternative exon skipping (CD19 exon 2 skipping)	CD19 exon 2 skipping → loss of binding epitope; trogocytosis → reduced antigen density on leukemia	Multi-antigen targeting (CD19/CD22 bispecific CARs); sequential targeting	~10–15% of relapses after CD19-directed therapy	[27]
T-cell exhaustion	Progressive dysfunction of CAR T-cells due to chronic antigen stimulation	NFAT → TOX → upregulation of PD-1, TIM-3, LAG-3 → reduced proliferation and cytokine production	Checkpoint inhibition (PD-1/PD-L1 blockade); armored CARs with cytokine secretion	Common in relapsed disease after prior immunotherapy	[28]
Lineage switch	B-ALL converts to myeloid or mixed phenotype acute leukemia under selective pressure	KMT2A rearrangement; epigenetic reprogramming → loss of B-cell markers (CD19, CD22)	Target alternative antigens (CD33, CD123); switch to myeloid-directed therapy	5–10% of CAR T-cell relapses; poor prognosis	[29]
Immunosuppressive microenvironment	Accumulation of regulatory T-cells (Tregs), myeloid-derived suppressor cells (MDSCs), and inhibitory cytokines	TGF-β, IL-10, IL-35 secretion; hypoxia (HIF-1α upregulation); extracellular matrix barriers	Armored CARs with IL-15 secretion; oncolytic viruses; ECM-degrading enzymes (hyaluronidase)	Major barrier in solid tumors (neuroblastoma, osteosarcoma)	[30]
Pharmacokinetic failure	Insufficient drug exposure due to poor penetration or rapid clearance	Large antibody constructs (e.g., blinatumomab) have limited CNS penetration; short half-life	Continuous intravenous infusion (blinatumomab); locoregional delivery (intrathecal, intraventricular)	CNS relapse despite systemic response (AALL1731 trial)	[31]
Sanctuary site involvement	Leukemic cells protected in CNS or testes where drug penetration is limited	Blood–brain barrier; blood-testis barrier	Intrathecal chemotherapy; cranial radiation (select cases); intensified systemic therapy	CNS relapse unchanged by blinatumomab in AALL1731	[32]

**Table 4:** Toxicity Burden of Cancer Therapy as a Determinant of Survival Outcomes.

Domain	Toxicity Type	Mechanisms/Causes	Clinical Manifestations	Impact on Treatment & Survival	Key Data/Notes
Short-term (Acute)	Infectious complications	B-cell depletion, neutropenia, central venous catheters	Sepsis, catheter-related infections	Dose reduction, treatment interruption, mortality	14.8% vs. 5.1% infection rate in combination therapy
	Immune activation toxicity	T-cell activation	Cytokine Release Syndrome (CRS): fever, hypotension, respiratory insufficiency	May require tocilizumab or corticosteroids; risk of life-threatening events	Usually mild–moderate but potentially fatal
	Neurologic toxicity	CNS immune effects, T-cell trafficking	Headache, tremor, somnolence, seizures	Temporary treatment discontinuation	Generally reversible
	Conventional chemotherapy toxicity	Damage to rapidly dividing cells	Myelosuppression, mucositis, organ toxicity	Dose-limiting toxicities	11.2% dose modification; 9.0% DLT in phase I trials
	Targeted therapy toxicity	Signaling pathway interference	Hypertension, cardiac dysfunction, metabolic disorders	Defines maximum tolerated dose	Mechanism-based adverse effects
	Immunotherapy toxicity	Immune system overstimulation	ICANS, autoimmune phenomena	Requires intensive monitoring	Organ-specific inflammatory damage
Long-term (Late Effects)	Neurocognitive impairment	Cranial radiation, intrathecal chemotherapy	Memory, attention, executive dysfunction	Impaired education and employment	Common in CNS-directed therapy
	Fertility impairment	Alkylating agents, radiation	Infertility	Psychosocial burden	Affects family planning
	Growth abnormalities	Cranial or total body irradiation	Short stature, GH deficiency	Hormone replacement therapy	Lifelong endocrine follow-up
	Cardiopulmonary toxicity	Anthracyclines, bleomycin, radiation	Cardiomyopathy, pulmonary fibrosis	Increased morbidity and mortality	Organ-specific damage
	Renal/hepatic toxicity	Cisplatin, ifosfamide, methotrexate	Nephropathy, hepatopathy	Chronic disease risk	Lifelong surveillance required
	Endocrine dysfunction	Radiation exposure	Thyroid, adrenal, gonadal disorders	Hormonal replacement therapy	Multisystem involvement
	Secondary malignancies	Radiation, genetic predisposition	Therapy-related leukemia, solid tumors	Increased cancer risk	Most feared late effect

## Data Availability

No new data were created or analyzed in this study. Data sharing is not applicable to this article.
